# Navigating Fish Immunity: Focus on Mucosal Immunity and the Evolving Landscape of Mucosal Vaccines

**DOI:** 10.3390/biology13120980

**Published:** 2024-11-27

**Authors:** Mai G. Hopo, Mahmoud Mabrok, Nermeen Abu-Elala, Yongyao Yu

**Affiliations:** 1 College of Fisheries, Huazhong Agricultural University, Wuhan 430070, China; vetmai1994@gmail.com; 2 Department of Fish Diseases and Management, Faculty of Veterinary Medicine, Suez Canal University, Ismailia 41522, Egypt; dr.mahmoudmabrok@yahoo.com; 3 Department of Microbiology and Parasitology, Faculty of Veterinary Medicine, King Salman International University, Ras Sudr 46612, Egypt; 4 Department of Aquatic Animal Medicine and Management, Faculty of Veterinary Medicine, Cairo University, Cairo 12211, Egypt; 5 Department of Animal Medicine, Faculty of Veterinary Medicine, King Salman International University, Ras Sudr 46612, Egypt; 6 Hubei Hongshan Laboratory, Wuhan 430070, China

**Keywords:** fish immune system, mucosal immunity, bacterial disease, vaccine, aquaculture

## Abstract

The growing role of aquaculture in global food security has underscored the need for advanced immunological insights to protect fish health and boost productivity. As aquaculture’s importance rises, understanding fish immunity is crucial for developing effective vaccination strategies. Fish possess a specialized immune system with unique mucosal structures that enable resilience in aquatic environments. This review examines critical advances in fish mucosal immunity, particularly focusing on mucosal vaccines that target infection at primary entry points, such as the gills, skin, and gastrointestinal tract. Mucosal vaccination has demonstrated a compelling capacity to stimulate localized and systemic immune responses, offering enhanced protection against waterborne pathogens. Additionally, this review addresses knowledge gaps from previous research on the global aquaculture vaccines market by offering a regional perspective on industry developments, recent trends, and innovative vaccine formulations. In doing so, it highlights the role of mucosal vaccines in overcoming the specific challenges of fish farming and supporting sustainable aquaculture. This synthesis of current methodologies, industry practices, and future directions contributes to a deeper understanding of fish immunology, ultimately informing strategies to achieve optimal disease management and bolster global aquaculture resilience.

## Simple Summary

This review article depicts the fish immune system and vaccination, focusing on mucosal vaccines. It underscores the distinctive features of the fish immune system, including mucosal and systemic immune responses that serve as defenses against various types of pathogens. The article further explores the different types of available fish vaccines. Mucosal vaccines are highlighted for their capacity to elicit robust local and systemic immune responses, enhancing disease resistance and promoting overall fish health in addition to significantly contributing to the overall health and well-being of fish populations, making them a crucial element in aquaculture and fisheries management.

## 1. Introduction

Aquaculture is recognized as a critical solution for addressing global food shortages, with the potential to bridge gaps in food security [1]. Despite its status as one of the fastest-growing sectors in agrifood, the sustainability of aquaculture is increasingly threatened by disease outbreaks, often exacerbated by intensified farming practices [2]. These practices are driven by rising consumer demand, urbanization, and expansion of aquaculture facilities, which have led to overcrowded environments, escalating the risk of infections and pathogen transmission [3]. Consequently, antibiotics are commonly used to control pathogens in aquaculture; however, their overuse has contributed to the rise of multidrug-resistant bacteria, posing a risk to fish health and public safety [4]. The global increase in multidrug resistance poses a significant public health threat. Recent studies have underscored the emergence of multidrug-resistant (MDR) aquatic pathogens, highlighting the urgent need for appropriate antibiotic use [5,6,7,8]. In response, vaccination has emerged as the preferred method for disease prevention in aquaculture, providing a safer alternative to antibiotics [9]. The vaccine can be formulated as a suspension of attenuated or killed microorganisms, toxins, or other biological agents containing protein or polysaccharide antigens, designed to provoke a protective immune response against the pathogen causing infection [10]. Advances in vaccine technology, including subunit, recombinant, and RNA-based vaccines, have improved fish health and disease resistance across multiple aquaculture species. However, while these innovations have been beneficial, current vaccine strategies often fall short in stimulating robust immunity at the mucosal surfaces, the primary entry points for many pathogens, including the gills, skin, and gastrointestinal tract [11]. Mucosal vaccines, therefore, present a promising solution by targeting these specific immune barriers, potentially enhancing protection against waterborne pathogens. Recent literature, including comprehensive reviews has advanced our understanding of immunoglobulins and mucosal immunity in teleost fish, providing insights into the complexities of fish immune responses [12]. Nevertheless, several knowledge gaps persist, particularly concerning the practical application of mucosal vaccines in aquaculture settings. Prior studies have focused on systemic immunity, overlooking the unique challenges that complicate vaccine delivery and efficacy in the aquatic environment. There remains limited information on optimizing mucosal vaccines for fish, including effective delivery methods and adjuvants that can withstand aquatic conditions [9]. This review addresses these gaps by exploring recent advancements in mucosal immunity and highlighting novel mucosal vaccination strategies tailored to fish. We evaluate current innovations and practical challenges in vaccine development, offering a comparative analysis of delivery methods suited for large-scale applications, such as immersion and oral vaccines that could improve disease resistance in fish farming. Through this focused review, we aim to fill critical knowledge gaps and provide a comprehensive understanding of the current state and perspective potential of mucosal vaccines in supporting sustainable aquaculture.

## 2. Key Characteristics of the Fish Immune System

Fish are exposed to a microbial-rich aquatic environment from the embryonic stage, interacting with both saprophytic and pathogenic microorganisms that can cause tissue damage. Under normal conditions, the fish immune system—a complex defense mechanism—allows fish to maintain health against these microbial invaders [13,14]. Fish represent a key group of lower vertebrates and provide essential insights into the early evolution of vertebrate immunity, making them valuable models for comparative immunological studies [15]. Osteichthyes, one of the earliest diverging vertebrate lineages, display both innate and adaptive immunity [16], which protects the body by preventing microbial attachment, inhibiting pathogen invasion, and managing inflammatory responses and tissue repair during infections [17].

### 2.1. Unique Features of Fish Immune Anatomy

The fish immune system differs from that of higher vertebrates, particularly in the absence of bone marrow and lymphatic nodules [18]. Instead, fish rely on the anterior (head) kidney as the primary lympho-hematopoietic organ, which is essential for B cell development [19]. The thymus is responsible for T cell maturation, while the spleen acts as the main secondary lymphoid tissue [20]. The fish immune system is composed of innate (nonspecific) and adaptive (specific) immunity, both featuring cellular and humoral components.

### 2.2. Innate Immune Response

Innate immunity is the primary defense mechanism in fish, providing immediate, nonspecific responses to infections. The innate immunity can either halt or control microbial attacks until a more efficient adaptive immune response develops [21]. This system includes physical barriers: The skin, mucosal surfaces (such as the skin, gills, and gastrointestinal tract), and mucus layer serve as essential barriers against pathogens. These barriers prevent the entry of microbial invaders, with mucus containing various immune molecules, including antimicrobial peptides and immunoglobulins, that facilitate both innate and adaptive immune responses [22,23]. Mucosal peptides specifically inhibit bacterial and fungal growth, enhancing protection against aquatic pathogens [24].

Complement System: This group of blood proteins is activated in response to pathogens, enhancing pathogen clearance and promoting inflammation. Once activated, complement cascades recruit macrophages and neutrophils to the infection site, which in turn attack pathogens through phagocytosis, coagulation, encapsulation, and the release of reactive oxygen and nitrogen species, which are critical for pathogen elimination [25,26].

Immune Cells: Macrophages and neutrophils play a key role in recognizing and engulfing foreign invaders [27]. If pathogens bypass these physical barriers, fish initiate cellular immune responses involving neutrophils, macrophages, and lymphocytes to eliminate these invaders [28]. These cells possess sophisticated receptor systems, such as Pattern Recognition Receptors (PRRs), which detect pathogen-associated molecular patterns (PAMPs) and signal the presence of microbial infections [29,30]. Through PRRs—such as Toll-like receptors (TLRs), NOD-like receptors (NLRs), C-type lectin receptors (CLRs), and peptidoglycan recognition proteins (PGRPs)—fish identify pathogen-associated molecular patterns (PAMPs) such as lipopolysaccharide (LPS), flagellin, and peptidoglycan [31]. These receptors perform specific functions, including opsonization, complement activation, and phagocytosis [32]. Their activation initiates immune pathways that enhance pathogen clearance, stimulate inflammatory responses, and encourage the release of pro-inflammatory mediators. For instance, LPS from Gram-negative bacteria induces cytokine release (e.g., TNFα, IL-6, IL-1β) to promote inflammation [33]. Peptidoglycans from Gram-positive bacteria trigger immune responses via PGRPs, leading to phagocytosis and the production of cytokines and prostaglandins [34]. Flagellin, a ligand for TLR5, activates the NF-κB pathway, enhancing immune responses to motile bacterial pathogens [35,36]. Research has demonstrated that these PRR pathways regulate fish immune responses across various species, with specific genes being activated following pathogen exposure, such as C-type lectins in *Senegalese sole* and TLRs in rainbow trout [37,38,39].

Additionally, fish rely on humoral components such as complement proteins, cytokines, and lysozymes in plasma and other body fluids to regulate immune responses and contain infections [40]. While some humoral components in fish share similarities with those in mammals, others are uniquely adapted for survival in aquatic environments, facilitating rapid responses to infections. Overall, the innate immune response in fish is crucial for their survival in diverse aquatic environments, allowing them to respond rapidly to infections.

### 2.3. Adaptive Immune Response

The adaptive immune system in fish, while similar in function to that of higher vertebrates, has unique characteristics [41]. It is primarily mediated by lymphocytes, including B cells and T cells: B Cells: These produce antibodies specific to pathogens, providing long-term immunity after initial exposure [42]. T Cells: T cells coordinate the immune response and can directly kill infected cells [43]. Fish-specific anatomical structures, such as the head kidney and spleen, create specialized microenvironments for B and T cells. These organs limit the available immune repertoires and regulate the initiation of adaptive immune responses [44].

The systemic immune response in fish involves a network of immune cells and molecules that work together to recognize, engulf, and destroy pathogens [41]. Additionally, fish produce a range of immune proteins, including cytokines, which help regulate immune activity and promote inflammation to combat infections [42]. The systemic response is supported by the complement system, enhancing pathogen clearance and facilitating the recruitment of more immune cells to the site of infection [45]. While it was originally believed that fish possessed only the immunoglobulin isoform IgM, recent studies have identified additional immunoglobulins, including IgD, IgZ, and IgT [46,47]. IgT and IgT+ B cells are now known to play predominant roles in mucosal immunity, contributing to the defense of mucosa-associated lymphoid tissues (MALTs) [12].

### 2.4. Mucosal Immune Response

**Figure 1:**
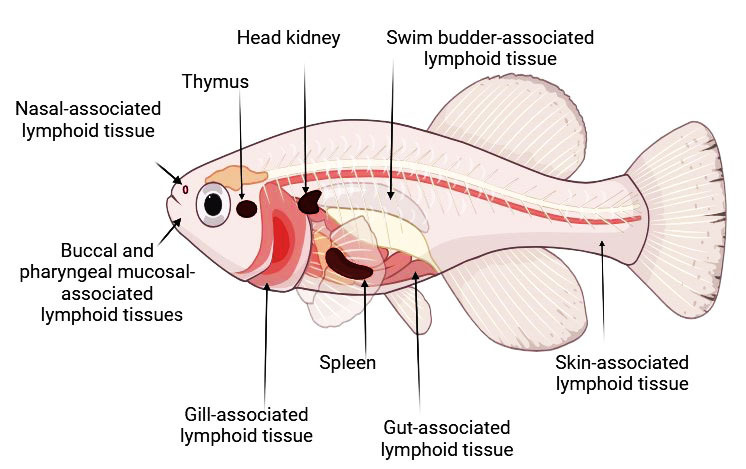
A diagram illustrating the immune organs and mucosal-associated lymphoid tissues in fish.

Mucosal immunity is essential for protecting fish against pathogens, especially at their mucosal surfaces—skin, gills, and intestines. Fish mucosal tissues are covered by a mucus layer that contains antimicrobial peptides, immunoglobulins, and enzymes to neutralize pathogens [48]. Specialized immune cells, such as goblet cells and lymphocytes, are present in these tissues to respond to invaders [49]. Fish possess several mucosa-associated lymphoid tissues (MALTs), including gut-associated lymphoid tissue (GALT), skin-associated lymphoid tissue (SALT), nasal-associated lymphoid tissue (NALT), buccal-associated, pharyngeal-associated, and swim bladder-associated lymphoid tissues [12,50]. These tissues are situated at key points of pathogen exposure (Figure 1).

Specifically, the intestinal mucosa comprises two main layers: the epithelial layer and the underlying lamina propria. Granulocytes, macrophages, and other leukocytes, including immunoglobulin-positive (Ig+) cells, are predominantly found in the lamina propria [51]. Interestingly, Ig+ cells are also present in the epithelial layer of the midgut and hindgut [52]. T cells are primarily located in the epithelial layer, while B cells reside in the lamina propria, both contributing to the fish’s mucosal immune defense [53]. In particular, immunoglobulin T (IgT) is a key player in fish mucosal immunity and is more abundant in the intestinal mucus compared to IgM. IgT is also more efficient at facilitating the phagocytosis of bacteria. Both IgM+ and IgT+ cells are distributed throughout the intestine, with IgM+ cells concentrated in the lamina propria and IgT+ cells mainly present as intraepithelial lymphocytes [12].

Mucosal surfaces, directly exposed to environmental threats, serve as the first line of defense through a dynamic network of immune factors that coordinate innate and adaptive responses to counter pathogens and maintain overall health [54]. The mucus layer covering these surfaces acts as a physical barrier, while immune molecules such as lysozymes, complement proteins, proteases, and antiproteases play a critical role in defending against invading pathogens, especially Gram-negative bacteria. For example, a time-course study by Guardiola, et al. [55] demonstrated that Senegalese sole (*Solea senegalensis*) exhibited a delayed mucosal immune response after being challenged with *Tenacibaculum maritimum*. Although early stages of infection showed weak bactericidal activity in the skin mucus, later stages revealed a significant increase in immune factors, suggesting a gradual but effective immune response, Mabrok, et al. [56]. Mucosal B cells, particularly those producing IgT and IgM antibodies, play a pivotal role in protecting fish against bacterial pathogens [57]. The nature of the infection, whether through immersion or injection, influences the type of immunoglobulin response. For example, rainbow trout (*Oncorhynchus mykiss*) infected with *Yersinia ruckeri* via immersion exhibited a marked increase in IgT levels in the intestinal mucosa, whereas IgM levels predominated in systemic tissues following injection [58]. Similarly, immersion with *Edwardsiella tarda* triggered higher IgT expression in the mucosal tissues of flounder (*Paralichthys olivaceus*), while systemic responses favored IgM production [59]. This underscores the specialization of IgT in mucosal immunity and IgM in systemic responses. Several studies highlight the dominant role of IgT in mucosal immunity.

In rainbow trout infected with *Flavobacterium columnare*, IgT+ B cells significantly increased in all mucosal tissues, with high levels of pathogen-specific IgT secreted into the mucus [60,61]. This increase in IgT was accompanied by a corresponding rise in IgM+ B cells in systemic tissues such as the spleen and head kidney, further emphasizing the distinct roles of IgT and IgM in mucosal and systemic immunity, respectively [62]. However, recent findings suggest that IgM may also play a role in mucosal defense. Mu, et al. [63] found high levels of specific IgM in both the serum and intestinal mucus of carp infected with *Aeromonas hydrophila*. Similarly, von Gersdorff Jørgensen, et al. [64] reported an increase in IgM+ and IgD+ B cells in the skin of rainbow trout infected with *Flavobacterium psychrophilum* in a cohabitation model. These results indicate that both IgT and IgM contribute to mucosal immunity, with their expression levels varying depending on the pathogen and mode of infection. In addition to the role of B cells, T cells are critical for clearing bacterial infections. For example, helper T cells and cytotoxic T lymphocytes have been shown to be essential for protection against *Streptococcus agalactiae* and *E. tarda* in tilapia, respectively [65,66]. T cells contribute by regulating the immune response and directly eliminating infected cells, thereby supporting both mucosal and systemic immunity. Overall, fish mucosal immunity is a sophisticated system capable of mounting targeted responses against bacterial pathogens. The interplay between mucosal B cells, immunoglobulin types (IgT and IgM), and T cells ensures a robust defense, highlighting the complexity and specialization of immune responses in different tissues and against various pathogens.

## 3. Overview of Aquatic Vaccines

Aquatic vaccine research, initiated in the mid-20th century, has yielded significant advancements in aquaculture prevention and control of bacterial and viral diseases. A substantial increase in commercially available vaccines has occurred since the 1980s; however, their efficacy and accessibility remain variable across different fish species [67,68,69]. Despite progress, several critical challenges persist. Developing effective vaccines for parasitic infections lags behind bacterial and viral counterparts [70]. Furthermore, the predominant administration method, intraperitoneal injection, is suboptimal for large-scale aquaculture operations due to its associated costs and operational complexities [71]. Economic constraints often necessitate using antimicrobials over-vaccination, contributing to antimicrobial resistance. To address these limitations, future research should prioritize the development of mucosal vaccines and innovative delivery systems. Additionally, a comprehensive understanding of pathogen similarities can facilitate the creation of broadly protective vaccines.

The aquaculture industry can achieve sustainable growth through improved disease prevention and control by overcoming these obstacles. One promising avenue is the development of genetically engineered vaccines. Recombinant vaccines represent a significant leap forward in vaccine design [72]. Researchers can produce large quantities of the desired antigen in a controlled environment by introducing the genetic code for a specific pathogen antigen into a host organism (bacteria, yeast); this approach minimizes the risk of unwanted immune responses to irrelevant bacteria or viral components. Recombinant production methods enable the rapid and consistent manufacture of large vaccine batches, which is crucial for meeting the demands of the aquaculture industry [73]. Recombinant antigens are highly purified, ensuring vaccine safety and minimizing side effects.

Alternatively, nanoparticles can shield antigens from degradation and enhance their uptake by immune cells, resulting in a stronger immune response [74]. Nanotechnology offers a revolutionary approach to vaccine delivery. Scientists can enhance vaccine efficacy and functionality by encapsulating antigens in tiny particles (particles ranging from 1–100 nanometers in size) [75,76]. They can be tailored to target specific tissues or cell types, optimizing vaccine efficacy while reducing off-target effects. Furthermore, nanoparticles can be engineered for slow antigen release, ensuring a more sustained immune response [77,78]. For example, studies have shown that encapsulating antigens in nanoparticles designed to overcome mucosal barriers (common entry points for aquatic pathogens) can significantly improve vaccine efficacy in fish.

Meanwhile, omics technologies provide invaluable insights into the genetic makeup of both pathogens and fish. By understanding the molecular basis of disease, researchers can identify novel vaccine targets and design more effective immunogens. Furthermore, computational modeling accelerates vaccine development by predicting antigen structure and immune responses. While these advancements hold immense promise, challenges such as cost, regulatory hurdles, and the need for extensive validation must be overcome. Nonetheless, the benefits of these technologies in disease prevention, enhanced fish health, and economic sustainability for the aquaculture industry are undeniable. As research progresses and these innovations mature, we can anticipate a future where aquaculture is more resilient to disease outbreaks and capable of meeting the growing global demand for seafood.

## 4. Global Aquaculture Vaccines Market: Regional Analysis and Key Players

The global aquaculture vaccine market is experiencing significant growth due to increased aquaculture production, higher disease prevalence, and a growing emphasis on sustainable practices [79,80]. Europe is a dominant force in the market, with its strong aquaculture sector, supportive government policies, and robust research infrastructure promoting vaccine development [81]. North America’s strong regulatory framework and commitment to combating antimicrobial resistance contribute to market expansion. The Asia-Pacific region is witnessing rapid expansion, with abundant water resources, supportive government policies, and a rising middle class increasing protein consumption. Latin America faces challenges related to disease outbreaks, emphasizing the need for effective vaccines. The Middle East and Africa are emerging markets for aquaculture vaccines, driven by government support and private investments. The region’s focus on environmentally friendly and technologically advanced aquaculture aligns with the growing importance of vaccines in disease prevention and control. As the aquaculture industry expands, vaccine demand is expected to rise significantly (Aquaculture Vaccines Market, https://straitsresearch.com/report/aquaculture-vaccines-market, accessed on 23 October 2024).

The aqua vaccines market is a competitive industry with key players such as Zoetis and Merck (MSD) driving innovation and market growth. These companies have extensive research and development capabilities, catering to various aquaculture species and markets worldwide. Regional companies such as HIPRA, Tecnovax, and Virbac also have substantial market shares. HIPRA has a strong presence in European and Latin American markets, while Tecnovax specializes in cold-water species vaccines. Virbac, a French multinational company, offers a broad spectrum of animal health products, including aqua vaccines (Aquaculture Vaccines Market Outlook 2023 to 2033, https://www.futuremarketinsights.com/reports/aquaculture-vaccines-market, 12 August 2024). The aquaculture vaccine market size surpassed USD 214.6 million in 2022 and is expected to witness a 7.6% CAGR between 2023 and 2032 (Aquaculture Vaccines Market Size, https://www.gminsights.com/industry-analysis/aquaculture-vaccines-market, 12 August 2024).

## 5. The Present Commercially Available Aquaculture Vaccines

The commercial aquaculture industry relies heavily on vaccines to protect fish and other aquatic organisms from a wide range of pathogens, ensuring both health and productivity in operations. The first commercially available fish vaccines were developed in 1980 to combat vibriosis and enteric red mouth disease (ERM), followed by vaccines targeting furunculosis [82]. Today, over 50 vaccines are tailored to more than 30 fish species. These vaccines have received approval from the United States Department of Agriculture (USDA) and include inactivated (killed) vaccines, live attenuated vaccines, subunit vaccines, DNA vaccines, and recombinant vector vaccines. Each type of vaccine serves a specific purpose and has distinct commercial applications. The primary types of commercially available vaccines are:

### 5.1. Inactivated Vaccines

Whole-cell inactivated vaccines represent the predominant vaccination strategy employed within the aquaculture industry [83,84]. A list of the most commercially available inactivated bacterial and viral vaccines in the aquaculture sector is provided in (Table 1). However, only one commercially available parasitic vaccine for sea lice in salmonids (Providean Aquatec Sea Lice) was introduced in 2015, produced by Tecnovax S.A. Argentina [70]. These vaccines are produced by inactivating viable pathogens through chemical or physical treatments, such as formalin, heat, or radiation. This process eliminates the pathogen’s virulence while preserving its antigenic properties, thereby inducing an immune response in the host [85]. While offering enhanced safety, whole-cell inactivated vaccines typically exhibit lower immunogenicity than other vaccine types. Consequently, adjuvants or multiple booster doses are often necessary to achieve sufficient protection. Furthermore, targeting heterogeneous pathogens to optimize vaccine performance can compromise the vaccine’s efficacy. Careful consideration of both host and pathogen characteristics is essential. The technology’s limitations extend to intracellular and viral pathogens, restricting its applicability to a subset of bacterial agents [9]. Despite these drawbacks, whole-cell inactivated vaccines remain a cornerstone of aquaculture vaccination due to their affordability, straightforward production, and robust stability during storage and transportation [84]. These attributes have facilitated widespread adoption and application in the industry. As such, they provide effective protection against many aquatic diseases.

The table data are sourced from the previous publication [79,84,86].

### 5.2. Live Attenuated Vaccines

Live-attenuated vaccines constitute a second major category of commercially available aquaculture vaccines (Table 2). These formulations contain live pathogens that have been weakened through a process that reduces their virulence while maintaining their ability to trigger an immune response. Various methods, such as chemical or physical treatments, serial passaging, or genetic engineering, can achieve attenuation [83]. Unlike inactivated vaccines, live-attenuated vaccines replicate within the host, simulating natural infection and eliciting robust humoral and cellular immune responses. This characteristic often results in longer-lasting immunity and reduced reliance on adjuvants or booster vaccinations [85,87]. However, the use of live pathogens carries inherent risks, including the potential for reversion to virulence and contraindication in immunocompromised individuals [88]. Rigorous strain selection, characterization, and attenuation are crucial for mitigating these risks. Producing and handling live-attenuated vaccines necessitates more complex technological infrastructure and stringent cold chain management than inactivated vaccines. While their efficacy is undeniable, the associated challenges underscore the need for continuous research into alternative vaccine platforms to address the evolving needs of the aquaculture industry.

### 5.3. Subunit Vaccines

Subunit vaccines represent a modern approach to vaccine development that leverages specific pathogen components to induce immune responses. Unlike whole-cell or live-attenuated vaccines, subunit vaccines eliminate the risk of infection while maintaining high specificity. This precision enables targeted immune stimulation and minimizes adverse effects [85,88]. Subunit vaccines are often produced using recombinant DNA technology, which allows for the expression of desired antigens in heterologous systems [85,88]. While this approach offers significant advantages, it can be resource-intensive and challenging to implement in economically constrained aquaculture settings. In addition, because subunit antigens are isolated, they may not be as effective at immunizing people as whole-cell or live-attenuated vaccines [84]. For the best protection, it is necessary to use adjuvants and administer multiple booster doses. Despite these challenges, subunit vaccines have shown promise in preventing specific aquatic diseases, particularly those caused by viral pathogens. Continued advancements in recombinant protein technology and adjuvant development may enhance the efficacy and cost-effectiveness of subunit vaccines in aquaculture. Table 3 provides a brief overview of some commercially available subunit vaccines.

### 5.4. DNA Vaccines

DNA vaccines represent a cutting-edge approach to vaccination in aquaculture. These vaccines stimulate protective immune responses by directly introducing genetic material encoding specific pathogen antigens into the host. They also stimulate the production of protective immune responses [9,84]. Expression vectors, typically plasmids, facilitate antigen gene delivery and expression within host cells [84,85]. DNA vaccines offer several advantages, including rapid production, high safety profiles, and the potential for broad-spectrum protection against various pathogens, particularly intracellular ones. Their ability to induce both cellular and humoral immune responses often results in robust and long-lasting immunity [89]. However, the potential for foreign DNA integration into the host genome remains a subject of ongoing investigation [90]. Additionally, consumer acceptance and regulatory hurdles have limited the widespread adoption of DNA vaccines in aquaculture [89]. Despite these challenges, recent commercial successes with DNA vaccines against Infectious hematopoietic necrosis virus (IHNV) (commercially known as Apex-IHN^®^, Elanco Co., Greenfield, IN, USA) and Salmonid alphavirus (SAV-3) (commercially known as ClynavTM, MSD, Rahway, NJ, USA) [79] have been used, highlighting the significant potential of this technology for improving disease control and aquaculture sustainability [9,85,91].

## 6. Emerging Trends in Aquaculture Vaccines

### 6.1. Messenger RNA Vaccines

These types of vaccines represent a promising and rapidly advancing technology with substantial potential for aquaculture [91,92]. They consist of mRNA molecules encoding specific pathogen antigens. Upon administration, the mRNA is translated into protein within the host cell, eliciting an immune response [93]. The key advantages of mRNA vaccines include rapid development, high immunogenicity, and a reduced risk of adverse events compared to traditional vaccine platforms. Additionally, the ability to produce large quantities of mRNA relatively quickly offers the potential for a rapid response to emerging pathogens. While conventional mRNA vaccines rely on transient expression of the encoded antigen, self-amplifying mRNA vaccines, based on viral replicates, can enhance antigen production and prolong immune responses [92,93]. Alphaviruses, a family of positive-sense RNA viruses, have been explored as platforms for developing self-amplifying mRNA vaccines due to their ability to replicate in a wide range of hosts, including fish [94]. Despite the significant potential of mRNA vaccines in aquaculture, several challenges remain, including mRNA stability and delivery methods. Hence, further research is needed to optimize immune responses in aquatic species. Nevertheless, early studies demonstrating the efficacy of mRNA vaccines against aquatic pathogens, such as infectious salmon anemia virus, highlight the promising future of this technology in the industry.

### 6.2. Vector Vaccines

Vector vaccines employ live, non-pathogenic microorganisms as carriers for delivering vaccine antigens [85,95]. This strategy combines the immunogenicity of live attenuated vaccines with the precision of subunit vaccines, offering a promising approach to aquaculture disease prevention [86,88]. A variety of bacterial species, including *Listeria monocytogenes*, *Escherichia coli*, *Bacillus subtilis*, *Lactobacillus casei*, and *Lactococcus lactis*, have been explored as vectors for delivering antigens against a range of aquatic pathogens [96,97]. These bacteria offer advantages such as ease of cultivation and genetic manipulation, making them attractive candidates for vaccine development. Baculoviruses, known for their ability to infect invertebrates, have been investigated as vectors for aquaculture vaccines, particularly against viral diseases affecting fish and shrimp [98,99,100,101,102,103]. However, the potential for genome integration and associated risks, including oncogenicity and GMO regulations, limit their commercial application [104]. On the other hand, adenoviruses exhibit a favorable safety profile and versatility as viral vectors, making them promising candidates for developing aquaculture vaccines. The advent of recombinant adenovirus technology has significantly advanced the field of aquaculture vaccinology. Successful applications in developing vaccines against bacterial pathogens such as *Aeromonas salmonicida* and viral pathogens such as IHNV and IPNV underscore the potential of this platform [105,106,107]. The ability to create multivalent vaccines, targeting multiple pathogens simultaneously, represents a significant step forward in disease prevention.

While vector vaccines offer significant potential, challenges such as vector safety, immune response modulation, and production costs should be addressed. Furthermore, the development of effective delivery systems and the optimization of antigen expression are crucial for the successful application of vector vaccines in aquaculture. Continued research into novel vector systems and the development of advanced genetic engineering techniques are essential for overcoming these challenges and realizing the full potential of vector vaccines in aquaculture disease prevention.

#### Synthetic Vector Vaccines

The emergence of synthetic vector platforms, such as virus-like particles (VLPs) and bacterial ghosts (BGs), offers alternative approaches to vaccine development. Virus-like particles (VLPs) are self-assembling protein structures that mimic viral particles without containing genetic material [108]. VLPs based on nodaviruses, particularly NNV, have shown promise as vaccine carriers against a range of viral and bacterial pathogens [108,109]. Their high immunogenicity, safety, and ability to induce both humoral and cellular immune responses make them attractive candidates for vaccine development. Bacterial ghosts (BGs) are derived from bacterial cell envelopes; BGs retain immunogenic components while lacking intracellular content. Their adjuvant properties and ability to stimulate both innate and adaptive immune responses make them potential vaccine carriers [110,111]. Applications against bacterial and viral pathogens have been explored, demonstrating their versatility. While VLPs and BGs offer promising avenues for vaccine development, several challenges remain. These include optimizing antigen presentation, ensuring efficient delivery, and overcoming production costs. Additionally, the regulatory landscape for synthetic vaccines requires careful consideration. Continued research into the development of novel vector platforms, as well as the optimization of existing ones, is essential for addressing these challenges and realizing the full potential of vector vaccines in aquaculture.

### 6.3. Synthetic Peptide or Epitope Vaccines

Synthetic peptide vaccines (epitope vaccines) are a more targeted approach to vaccine development, focusing on specific immunogenic regions of a pathogen. The advances in computational biology have enabled the identification and synthesis of these peptides, offering a precise and safe method for inducing protective immune responses. While still in its early stages in aquaculture [83,85], this technology has shown promise in targeting a variety of aquatic pathogens, including bacteria, viruses, and parasites. Efforts to identify and characterize epitopes for key pathogens such as *Edwardsiella tarda*, *Flavobacterium columnare*, and *Vibrio anguillarum* have laid the groundwork for epitope-based vaccine development [112,113]. The integration of immunoinformatics and proteomics has accelerated epitope discovery and vaccine design [114,115].

This, coupled with the potential of artificial intelligence, offers exciting prospects for the future of epitope vaccine development in aquaculture. By combining these advanced technologies with a deep understanding of aquatic organism immunology, it is anticipated that highly effective and specific vaccines can be created to address the complex challenges of aquatic disease prevention. The potential of synthetic peptide vaccines is promising, but there are still several challenges to address. For instance, ensuring that synthetic peptides trigger strong immune responses often requires the use of adjuvants or delivery systems. Additionally, many epitopes depend on specific protein structures, which can be challenging to mimic with linear peptides. Moreover, the synthesis of large quantities of peptides can be costly. It is important to address these challenges, especially the translation of synthetic peptide vaccines into commercial aquaculture applications through ongoing research and development.

## 7. Adjuvants in Aquaculture Vaccines: Current Trends, Mechanisms, Challenges, and Prospects

Adjuvants play a crucial role in enhancing the efficacy of vaccines by boosting the immune response. In aquaculture, where vaccines are key to controlling infectious diseases, adjuvants are particularly important due to the distinctive immune systems of fish and the aquatic environment. This review explores the different types of adjuvants used in aquaculture vaccines, their mechanisms of action, development and application, and potential future advancements.

### 7.1. Types of Adjuvants Used in Aquaculture Vaccines

A. Oil-based adjuvants

Oil-based adjuvants are the most commonly used in commercial fish vaccines. They include water-in-oil (W/O) emulsions and water-in-oil-in-water (W/O/W) emulsions, which prolong antigen exposure and enhance the immune response by creating a depot effect at the injection site. The Montanide ISA series, particularly ISA 763A, is widely used due to its ability to induce strong and long-lasting immune responses. These adjuvants have been effectively used in vaccines against various fish pathogens (Table 4). Freund’s Incomplete Adjuvant (FIA): Although effective, FIA is less commonly used in commercial vaccines due to its potential to cause severe inflammatory reactions, making it more suitable for experimental studies.

B. Aluminum-based adjuvants

Aluminum salts, such as aluminum hydroxide and aluminum phosphate, are commonly used adjuvants in human and veterinary vaccines. While less frequently used in fish, they have shown potential in boosting humoral responses when combined with other adjuvants. Alum enhances the uptake and presentation of antigens by immune cells, particularly promoting the production of antibodies [124]. It is often used alongside other adjuvants to improve the overall immune response in fish, especially in vaccines designed to combat viral diseases.

C. Saponin-based adjuvants

Saponins are natural glycosides known for their ability to enhance both humoral and cellular immune responses. Quil A, a purified saponin, has been utilized in experimental vaccines for fish species such as salmonids. It is particularly effective in inducing a balanced Th1/Th2 response, making it suitable for vaccines targeting intracellular pathogens [125].

D. Liposome-based adjuvants

Liposomes, spherical lipid bilayer vesicles, have emerged as promising carriers for antigen delivery in vaccine formulations. By encapsulating antigens, liposomes enhance antigen stability, protect them from degradation, and facilitate their uptake by immune cells. Liposomes have been tested experimentally on rainbow trout, carp, and zebrafish [126,127]. They are particularly promising for oral and immersion vaccines, where they protect antigens from degradation in the aquatic environment and gastrointestinal tract.

E. Nanoparticle-based adjuvants

Nanoparticles, including polymeric and chitosan-based particles, offer innovative platforms for vaccine delivery. They enhance immune responses by protecting antigens from degradation, facilitating targeted delivery to immune cells, and allowing for controlled antigen release. Poly (lactic-co-glycolic acid) (PLGA) nanoparticles have been used to enhance the immunogenicity of antigens in fish, showing promise in experimental vaccines for species such as carp and trout [128]. Chitosan nanoparticles, a biocompatible polymer, have been utilized in nanoparticle form for oral vaccines in species such as zebrafish and Atlantic salmon. These nanoparticles can adhere to mucosal surfaces, enhancing antigen uptake and immune response [129].

F. Immune-stimulating complexes

Immune-stimulating complexes (ISCOMs) represent an innovative approach to vaccine development [91]. These submicroscopic particles, typically less than 40 nanometers in diameter, are composed of antigen, cholesterol, and Quil A saponin. This unique combination creates a structure that effectively stimulates both humoral and cell-mediated immune responses. By encapsulating antigens within the ISCOM matrix, these particles enhance antigen presentation to immune cells, leading to a more robust immune response. Including Quil A saponin, a potent immunostimulant, amplifies the vaccine’s efficacy [130]. While ISCOM technology holds significant promise, further research is necessary to optimize its production, stability, and delivery methods. Additionally, studies on the long-term safety and efficacy of ISCOM-based vaccines are essential for their successful translation into clinical applications.

G. Cytokine adjuvants and immunostimulants

Cytokines such as interleukins (e.g., IL-1) and interferons (e.g., IFN-γ) enhance the immune response by activating immune cells, promoting inflammation, and improving antigen presentation [131]. Beta-glucans are polysaccharides that act as pathogen-associated molecular patterns (PAMPs), triggering the innate immune system and enhancing the overall immune response [132]. Numerous studies have affirmed that β-glucan, whether used alone or in combination with other active ingredients, is a valuable and promising immunostimulant for boosting immune function, improving growth performance, and managing diseases in fish farming [133,134,135]. Beta-glucans are already used in commercial fish feeds as immunostimulants, and their role as vaccine adjuvants is being explored in species such as tilapia [136] and catfish [137].

Cytokine adjuvants have been tested in experimental vaccines for several fish species, including Atlantic salmon [138], *Dicentrarchus labrax* [139], and carp [140], where they have shown promise in enhancing immune responses against viral and bacterial pathogens. Cytokines are proteins that can be unstable and difficult to deliver effectively in the aquatic environment. Maintaining their activity during storage and administration is a significant challenge. Producing recombinant cytokines or high-purity beta-glucans can be costly, limiting their widespread adoption as adjuvants. One promising approach is to use DNA or RNA vaccines that encode cytokines, allowing the fish to produce the adjuvant by themselves, which could enhance the immune response at lower costs [141]. Combining cytokines or beta-glucans with other adjuvants, such as nanoparticles or oil emulsions, may offer synergistic effects, leading to more robust and durable immune responses [142].

### 7.2. Mechanisms of Action

Adjuvants enhance vaccine efficacy through multiple mechanisms. Oil-based adjuvants and aluminum salts create a depot at the injection site, allowing for sustained antigen release and prolonged immune stimulation. This leads to a more robust and durable immune response. Additionally, adjuvants such as alum and nanoparticles facilitate antigen uptake by antigen-presenting cells, including dendritic cells and macrophages. This enhanced antigen presentation leads to more efficient T-cell activation and a more robust immune response. Furthermore, certain adjuvants, such as saponins and beta-glucans, stimulate the innate immune system. By activating innate immune receptors, these adjuvants trigger the release of cytokines, promoting both local and systemic immune responses. This synergistic combination of depot formation, antigen presentation enhancement, and innate immune stimulation contributes to the overall efficacy of vaccines [125,143].

### 7.3. Challenges in Adjuvant Development and Future Perspectives

While adjuvants are essential for enhancing vaccine efficacy, their application in aquaculture is complex due to several factors. Balancing the immunostimulatory properties of adjuvants with potential adverse effects, such as inflammation or granuloma formation, is critical for ensuring both fish health and product quality. The diverse immune systems of different fish species necessitate a tailored approach to adjuvant selection. Adjuvant performance is influenced by factors such as water temperature, salinity, and species-specific immune mechanisms. Moreover, the environmental impact of adjuvants, particularly oil-based emulsions, is a growing concern. The potential for adjuvant residues to enter aquatic ecosystems and harm non-target organisms requires careful consideration and ongoing research. Producing certain adjuvants, such as nanoparticles or recombinant proteins, can be expensive, limiting their widespread use in commercial vaccines. Advances in manufacturing technologies and formulation processes are needed to reduce costs and improve scalability. Developing safe and effective adjuvants for aquaculture remains a significant challenge that requires a comprehensive understanding of both immunological and environmental factors [86].

Developing novel adjuvants that are both effective and safe remains a priority in aquaculture vaccine research. This includes exploring new materials, such as biodegradable polymers and plant-derived adjuvants, which can enhance immune responses with minimal side effects. Combining different adjuvants to achieve synergistic effects is a promising approach. For example, combining oil-based adjuvants with nanoparticles or saponins could enhance both humoral and cellular responses, providing broader protection against pathogens. With the growing interest in mucosal vaccines, particularly for oral and immersion delivery, there is a need for adjuvants that can enhance immune responses at mucosal surfaces. Research into mucoadhesive nanoparticles, bioadhesive polymers, and other mucosal adjuvants is ongoing [144].

## 8. Mucosal Vaccines in Aquaculture

Mucosal vaccines represent a promising and innovative alternative to traditional injection-based vaccines in aquaculture [145]. These vaccines provide a non-invasive approach to stimulate robust immune responses at mucosal surfaces, including the gut, gills, and skin, the critical barriers against pathogens. By targeting these initial sites of infection, mucosal vaccines can effectively prevent pathogen replication and reduce disease outbreaks [11]. Despite challenges such as ensuring antigen stability, overcoming immune evasion mechanisms, and managing variable immune responses, significant progress has been made in developing advanced adjuvants, delivery systems, and vaccine formulations that enhance the efficacy and practicality of mucosal vaccines.

Several commercially available mucosal vaccines have been developed, with their applications tailored to specific aquaculture needs. For example, oral vaccines are widely used for their ease of administration, especially in large-scale operations, but they often face challenges with antigen degradation in the digestive system. On the other hand, immersion vaccines are commonly applied for bacterial infections such as vibriosis and furunculosis, offering consistent protection through absorption via the skin and gills. Nasal vaccines, while still in the early stages of development, show potential for generating localized immune responses in the nasal cavity, an important mucosal site.

Comparing their commercial use, oral vaccines stand out for their scalability and cost-effectiveness, particularly in large aquaculture farms. In contrast, immersion vaccines are preferred for their ability to deliver antigens without the need for specialized feeding protocols. Nasal vaccines, though less commonly applied commercially, are being investigated for their unique ability to stimulate both mucosal and systemic immunity. These advancements highlight the growing potential of mucosal vaccines to transform disease prevention strategies in aquaculture, with ongoing research into teleost fish B and T cell responses providing critical insights for future development [146]. Table 5 provides examples of some commercially available mucosal vaccines.

### 8.1. Delivery Methods for Aqua Mucosal Vaccines

**Figure 2:**
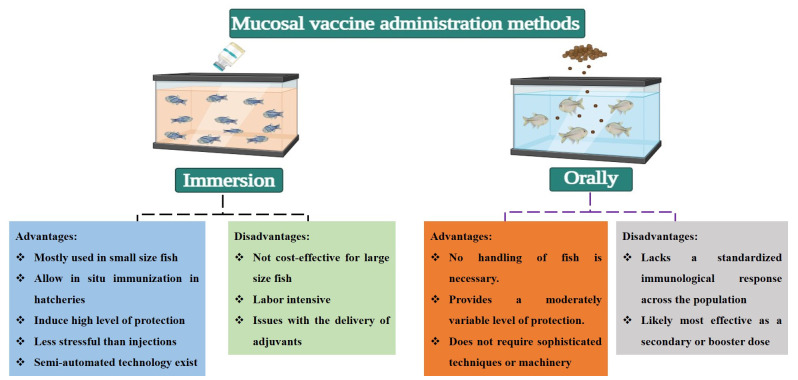
An overview of the different administration routes for mucosal vaccines, highlighting their respective advantages and disadvantages.

Immersion vaccination involves immersing fish in a solution containing the vaccine. It is particularly effective for small or juvenile fish and is commonly used in hatcheries [147]. This method allows antigens to contact the skin, gills, and possibly the gut mucosa, inducing a localized immune response [148]. The drawbacks of this method include its limited applicability for large fish in open sea cages, a shorter duration of immune response, and the potential variability in the dose received by each fish [149]. Additionally, antigen uptake via the immersion route is considerably affected by various factors, including the duration of vaccine exposure, total biomass, fish age, and the pH and salinity of the immersion water [150]. Oral vaccination involves incorporating vaccines into feed, allowing for easy mass administration. Microencapsulation and mucoadhesive nanoparticles represent innovative strategies to boost oral vaccine efficacy by shielding antigens from the harsh digestive environment and enabling their targeted release in the gut. This targeted delivery allows antigens to interact effectively with gut-associated lymphoid tissue (GALT), thereby inducing both systemic and mucosal immunity [86,151]. However, challenges such as antigen degradation in the digestive tract and variable uptake between individual fish limit the effectiveness of this method [11]. A summary of various administration routes for mucosal vaccines, along with their respective advantages and disadvantages, is provided in Figure 2.

### 8.2. Adjuvants Used in Aqua Mucosal Vaccines

Adjuvants are critical in mucosal vaccines to enhance the immune response. Adjuvants used in mucosal vaccines for fish include oil-based emulsions (OW emulsion) [147], bacterial components, nanoparticles, and cytokines. Oil-based adjuvants such as Montanide and particulate carriers such as liposomes and nanoparticles protect antigens from degradation and promote uptake by mucosal immune cells [152]. These adjuvants can also act as depots, slowly releasing the antigen and prolonging the immune response [153]. Saponins and beta-glucans are natural adjuvants that have shown promise in enhancing mucosal immune responses [154]. In particular, beta-glucans can activate innate immune receptors, producing cytokines that enhance both local and systemic immunity [91].

### 8.3. Immune Responses to Aqua Mucosal Vaccines

Oral immunization in fish triggers both local and systemic immune responses [11]. The hindgut is particularly important for antigen uptake, with larger antigens absorbed mainly by hindgut epithelial cells and smaller molecules passing through cell junctions [155]. Once absorbed, antigens are phagocytosed and transported via the bloodstream or lymphatic system to lymphoid organs, where they initiate an immune response. The hindgut is especially rich in immune cells, such as macrophages, granulocytes, and plasma cells, which play a key role in this process [156]. Lymphoid tissue, although not as organized as in mammals, has been identified in the intestines of some fish species, with a higher concentration of lymphocytes near the anus [157,158]. However, fish can experience oral tolerance, which is a diminished immune response to antigens administered orally. This phenomenon may be caused by factors such as high antigen doses, dysfunctional regulatory T cell (Treg) activity, antibody suppression, or repeated antigen exposure [159,160]. Other contributing factors include repeated low-dose antigen exposure, vaccinating young immunocompetent fish, low water temperatures, the characteristics of the antigen, the vaccination schedule, and genetic predispositions [161,162]. Since oral tolerance can negatively affect vaccine efficacy, it is essential to consider these factors carefully when designing oral vaccines for fish.

Growing evidence shows that mucosal vaccination can effectively stimulate both systemic and mucosal immune responses in fish, offering protection against pathogens [163,164]. In teleosts, three types of immunoglobulins have been identified: IgT/Z, IgM, and IgD. The immune responses involving IgM and IgT are key indicators of adaptive immunity following mucosal vaccination [48]. While IgM plays a critical role in systemic immunity, IgT is essential for mucosal immunity, functioning similarly to mammalian IgA [165]. Research by von Gersdorff Jørgensen, et al. [166] demonstrated that both IgM and IgT bind to the surface of *Ichthyophthirius multifiliis* in the gills of immune rainbow trout after infection. IgT+ B cells were predominantly located in the gill epithelia within the secondary lamellae, supporting findings from earlier studies [167]. Additional research by Wang and Dickerson [168] and Dickerson and Clark [169] showed that antibodies protect mucosal surfaces. In particular, antibodies from resistant fish were found to bind effectively to the immobilization antigens on parasite cells and ciliary membranes. These findings indicate that skin mucosa can generate both mucosal and systemic antibody responses following exposure to *I. multifiliis*.

In the gut of teleosts, IgT makes up the majority of the B cell population. Chen, Klaric, Wadsworth, Jayasinghe, Kuo, Evensen and Mutoloki [160] reported that when Atlantic salmon orally consumed alginate microspheres containing infectious pancreatic necrosis virus (IPNV), there was a significant increase in IgT transcription in the gut. Similarly, oral immunization with recombinant *Lactobacillus* expressing CK6 led to elevated β-defensin expression in the intestine and higher anti-VP2 IgT levels in skin mucus [170]. In rainbow trout vaccinated against *Yersinia ruckeri*, all three immunoglobulins (IgT, IgM, and IgD) were detected in the nasal mucosa, with IgT being the most prevalent [171]. These studies underscore the pivotal role of IgT in mucosal immunity following vaccination.

CD8α is recognized as a marker for cytotoxic T-lymphocytes (CTLs) in teleosts, representing a major component of the T-cell population in the gut [172,173]. Research suggests that CD8α+ T cells are activated via the MHC-I pathway. For instance, oral vaccination in grouper larvae (*Epinephelus coioides*) resulted in upregulation of MHC-I, which was associated with an increase in CD8α levels in the gut. [174]. Moreover, Martin, et al. [175] observed that natural cytotoxic activity in the intestinal leukocytes of rainbow trout was twice as high as in head kidney leukocytes, highlighting the importance of cellular immunity in the intestinal mucosa. Despite this, the mechanisms of cellular immunity in fish mucosal tissues remain underexplored.

Regarding CD4+ T cell responses, naïve CD4+ T cells differentiate into effector subtypes via the MHC-II pathway [161]. Several transcription factors involved in the differentiation of naïve CD4+ cells into specific T-helper (Th) cell types have been identified in various fish species [176]. Th cells play a crucial role in regulating the immune system by secreting specific cytokines. Zhang, et al. [177] found that both bath and injection vaccination methods triggered Th17-like responses in the mucosal immune system, while Th1 and Th2 responses were less prominent. Notably, bath vaccination induced stronger Th17-like immune responses in the gut tissue of zebrafish compared to injection vaccination. In recent years, inactivated vaccines, which predominantly stimulate CD4+ T cells and humoral immune responses, have been a major focus of vaccine research. However, there remains a challenge in developing effective delivery systems for intracellular antigens that can trigger both CD4+ and CD8+ T cell responses.

### 8.4. Challenges in Aqua Mucosal Vaccine Development

Oral administration is the most feasible delivery method, but the harsh environment of the fish gut (enzymes, low pH) can degrade antigens before they reach immune cells [178]. Immersion and spray methods are practical for large populations, but the challenge is to achieve consistent and effective antigen uptake across all fish. Compared to mammals, the fish mucosal immune system is less understood. Optimizing vaccine design requires a deeper understanding of antigen presentation, immune cell activation, and antibody production at mucosal sites. Immune responses can vary significantly between fish species, hindering the development of broadly effective vaccines. Developing formulations that remain stable in water and potent throughout the digestive process is crucial for oral vaccines. Adjuvants and delivery systems must be tailored for the aquatic environment to protect antigens and enhance immunogenicity. The cost-effectiveness of mucosal vaccines compared to injectable options requires careful analysis. The regulatory framework for mucosal fish vaccines is still evolving, posing challenges in obtaining market approval [179].

### 8.5. Future and Prospective Promises for Aqua Mucosal Vaccines

Developing effective mucosal vaccines for aquaculture requires a multifaceted approach encompassing antigen delivery, mucosal immunology, vaccine formulation, and efficacy evaluation [79]. Optimizing antigen delivery is crucial for vaccine success. Particle-based systems and mucoadhesive polymers can enhance antigen stability and mucosal retention [180]. The strategic selection of adjuvants can significantly boost immune responses [91]. A deep understanding of mucosal immunology, including immune cell distribution and function, is essential for designing targeted vaccines [86]. Developing multivalent vaccines that protect against multiple pathogens is a key goal, requiring careful antigen selection and formulation. Rigorous evaluation of vaccine efficacy through challenge studies and immune response monitoring is indispensable [181]. By integrating these strategies and conducting comprehensive research, the aquaculture industry can develop highly effective mucosal vaccines to safeguard fish health and promote sustainable production.

## 9. Conclusions

Fish immunity is a vital aspect of maintaining the health and sustainability of aquaculture. With a dual defense mechanism comprising innate and adaptive responses, fish are equipped to combat various pathogens. The introduction of vaccination has revolutionized aquaculture, significantly improving disease management and fish welfare. Effective vaccines boost the immune response, providing long-lasting protection against diseases, which is essential for the economic and biosecurity challenges of modern aquaculture. Advances in vaccine technology offer promising avenues for enhancing effectiveness and targeting diverse pathogens. Studying fish immunity is vital for vaccine evaluation, as it reveals how immune responses are activated after vaccination. Understanding the roles of antibodies and T cells helps researchers assess vaccine effectiveness and longevity of protection. This knowledge directly encourages the development of optimal vaccination strategies, resulting in more effective vaccines that improve fish health in aquaculture. Integrating vaccination strategies with optimal management practices will be crucial for achieving resilient fish populations. Ultimately, this comprehensive approach will not only fortify fish health but also contribute to the sustainable growth of the aquaculture industry.

## Figures and Tables

**Table 1:** List of commercially available inactivated bacterial, viral, and parasitic aqua vaccines (All websites are accessed on 20 August 2024).

Disease	Vaccine Acronym	Antigen	Delivery	Species
**(A) Inactivated commercially available bacterial vaccines**				
**Vibriosis**	ALPHA DIP^®^ Vib (Merck & Co., Inc., Rahway, NJ, USA) ALPHA DIP^®^ Vibrio PHARMAQ (https://pharmaq.com/en/pharmaq/our-products) (PharmaQ Co., Oslo, Norway)	*Listonella anguillarum* (serotype O1)	Immersion	Seabass
	AQUAVAC^®^ Vibrio MERCK-MSD (https://www.msd-animal-health.com/products/aquaculture/aquaculture-products/)	*L. anguillarum* (serotype O1) and *Vibrio ordalii* (strain MSC275)	Injection, Immersion, Oral	Trout, Seabass
	Icthiovac^®^ VR HIPRA (https://www.hipra.com/en/animal-health) (HIPRA Co. Girona, Spain)	*L. anguillarum* (serotypes O1, O2a, and O2b)	Injection, Immersion	Turbot
	Vibri-Fishvax^®^ FATRO (https://www.fatro.it/en/46-fish) (Farto Co., Ozzano dell’Emilia (BO), Italy)	*L. anguillarum* and *V. ordalii*	Injection Immersion	Salmonids, Seabass, Flatfish ayu
**Pasteurellosis**	AQUAVAC^®^ Photobac Prime MERCK-MSD	*Photobacterium damselae* subsp. *piscicida*	Immersion	Seabass, Seabream
	Icthiovac^®^ PD HIPRA	*Photobacterium damselae* subsp. *piscicida* (strains DI-21 and lt-1)	Immersion	Seabream
**Pasteurellosis and Vibriosis**	ALPHA DIP^®^ 2000 ALPHA JECT micro^®^ 2000 ALPHA JECT^®^ 2000 PHARMAQ	*L. anguillarum* (serotype O1) and *Photobacterium damselae* subsp. *piscicida*	Immersion Injection Injection	Seabass Seabass Seabass
	AQUAVAC^®^ Vibrio Pasteurella MERCK-MSD	*L. anguillarum* (Serotypes O1 and O2) and *Photobacterium damselae* subsp. *piscicida*	Injection	Seabass
	Icthiovac^®^ VR/PD HIPRA	*L. anguillarum* (Serotypes O1, O2a, and O2b) and *Photobacterium damselae* subsp. *piscicida* (strain DI21)	Injection	Seabass
**Streptococcosis**	ALPHA JECT micro^®^ 1 Tila PHARMA Q	*Streptococcus agalactiae*	Injection	Tilapia
	AQUAVAC^®^ Strep Sa MERCK-MSD	*S. agalactiae* (strain TI513)	Injection	Tilapia
	AQUAVAC^®^ Step Sa1 MERCK-MSD	*S. agalactiae* (Serotypes Ia and III)	Injection	Tilapia
	AQUAVAC^®^ Step Si MERCK-MSD	*Streptococcus iniae* (strain SB430)	Injection	Tilapia, Seabass
	Icthiovac^®^ STR HIPRA	*Streptococcus parauberis* (strains RA-99.1 and AZ-12.1)	Injection	Turbot
**Motile Aeromonas septicemia**	(2011) 190986013 Chinese Aquaculture Market [67]	*Aeromonas hydrophila*	Injection Immersion	Freshwater Fish
**Coldwater Vibriosis, Furunculosis, Vibriosis**	ALPHA JECT micro^®^ 4 PHARMA Q	*Aeromonas salmonicida* subsp. *salmonicida*, *L. anguillarum* (serotypes O1 and O2), and *V. salmonicida*	Injection	Atlantic salmon
	FORTE^®^ Micro ELANCO (https://www.elanco.com/en-us/products-and-services) (Elanco, Fort Dodge, IA, USA)	*A. salmonicida*, *L. anguillarum* (serotypes 1 and 2), *V. ordalii*, and *V. salmonicida* (serotypes 1 and 2)	Immersion	Salmonids
**Coldwater Vibriosis, Furunculosis, Vibriosis, Winter Sore**	ALPHA JECT micro^®^ 5 PHARMA Q	*A. salmonicida* subsp. *salmonicida*, *L. anguillarum* (serotypes O1 and O2a), *V. salmonicida*, and *Moritella viscosa*	Injection	Atlantic salmon
**Furunculosis, Vibriosis**	ALPHA JECT^®^ 3000 PHARMA Q	*A. salmonicida* subsp. *salmonicida* (strain AL2017) and *L. anguillarum* (serotypes O1 and O2a)	Injection	Salmonids
**Furunculosis, Pasteurellosis, Vibriosis**	ALPHA JECT^®^ 3 FPV PHARMA Q	*L. anguillarum* (serotype O1), *Photobacterium damselae* subsp. *piscicida* (strain AL5051), and *A. salmonicida* subsp. *salmonicida* (strain AL2017)	Injection	Seabass
**Winter Sore**	ALPHA JECT^®^ Moritella PHARMA Q	*M. viscosa* (strain AL21355)	Injection	Atlantic salmon
**Enteric septicemia, Motile Aeromonas Septicemia**	ALPHA JECT^®^ Panga 2 PHARMA Q	*Edwardsiella ictaluri* (strain AL20658) and *A. hydrophila* (serotypes A and B)	Injection	Pangasius
**Enteric Red Mouth** **(Yersiniosis)**	Alpha ERM Salar Alpha ERM Salar (Dip) PHARMA Q	*Yersinia ruckeri* (serotype O1b)	Injection Immersion	Atlantic Salmon
	AQUAVAC^®^ ERM MERCK-MSD	*Y. ruckeri* (Hagerman type 1 strain)	Injection Oral Immersion	Trout
	AQUAVAC^®^ YER MERCK-MSD	*Y. ruckeri* (Hagerman type 1 strain)	Injection	Atlantic salmon
	AQUAVAC^®^ RELERA MERCK-MSD	*Y. ruckeri* (Hagerman type 1 and EX5 biotype strains)	Injection Immersion	Trout
	Yersi-Fishvax^®^ FATRO	*Y. ruckeri* (Hagerman type 1 strain)	Injection Immersion	Salmonids
**Enteric Red Mouth, Vibriosis**	Bi-Fishvax^®^ FATRO	*L. anguillarum* and *Y. ruckeri*	Injection Immersion	Salmonids
**Lactococcosis**	Icthiovac^®^ LG HIPRA	*Lactococcus garvieae* (strain TW446.B3)	Injection	Trout
	Lacto-Fishvax^®^	*L. garvieae*	Injection	Trout
**Tenacibaculosis**	Icthiovac^®^ TM HIPRA	*Tenacibaculum maritimum* (strain LPV 1.7)	Injection	Turbot
**(B) Inactivated commercially available vaccines**				
**Infectious Salmon Anemia**	ALPHA JECT micro^®^ 1 ISA PHARMA Q	Infectious Salmon Anemia Virus (ISA) (strain ALV301)	Injection	Atlantic salmon
**Viral Nervous Necrosis**	ALPHA JECT micro^®^ 1 Noda PHARMA Q	Red Grouper Necrosis Virus (strain ALV1107)	Injection	Seabass
**Viral Nervous Necrosis**	Icthiovac^®^ VNN HIPRA	Betanodavirus (strain 1103)	Injection	Seabass
**Pancreas Disease**	ALPHA JECT micro^®^ 1 PD PHARMA Q	Salmon Pancreas Disease Virus (SPD) (strain ALV405)	Injection	Atlantic salmon
**Infectious Pancreatic Necrosis**	ALPHA JECT^®^ 1000 PHARMA Q	Infectious Pancreatic Necrosis Virus (IPNV) (strain ALV103)	Injection	Salmonids
**Iridoviral Infection Mortality**	AQUAVAC^®^ IridoV MERCK-MSD	Iridovirus	Injection	Tilapia, Seabass
**Grass Carp Hemorrhagic Disease**	Cell-cultured inactivated Grass Carp Hemorrhagic Disease Vaccine (1992) Chinese Aquaculture Market [67]	Grass Carp Reovirus	Injection	Carp
**Infectious Spleen and Kidney Necrosis**	Imported vaccine against fish Infectious Spleen and Kidney Necrosis (2014) Chinese Aquaculture Market [67]	Infectious Spleen and Kidney Necrosis Virus	Injection	Marine fish
**(C) Inactivated commercially available bacterial and viral vaccines**				
**Infectious Pancreatic Necrosis, Salmon Rickettsial Syndrome**	ALPHA JECT micro^®^ 2 PHARMA Q	IPNV (strain ALV013) and *Piscirickettsia salmonis* (strain AL10015)	Injection	Salmonids
**Infectious Pancreatic Necrosis Salmon, Rickettsial Syndrome, Vibriosis**	ALPHA JECT micro^®^ 3 PHARMA Q	IPNV (strain ALV103), *P. salmonis* (strain AL10015), and *V. ordalii* (strain AL510)	Injection	Atlantic salmon
**Furunculosis, Infectious Pancreatic Necrosis, Infectious, Salmon Anemia Vibriosis**	ALPHA JECT micro^®^ 4-2 PHARMA Q	*A. salmonicida* subsp. *salmonicida* (strain AL2017), IPNV (strain ALV103), and *V. ordalii* (strain AL510)	Injection	Atlantic salmon
**Coldwater Vibriosis, Furunculosis, Infectious Pancreatic Necrosis, Winter Sore**	ALPHA JECT micro^®^ 6 PHARMA Q	*A. salmonicida* subsp. *salmonicida*, *L. anguillarum* (serotypes O1 and O2a), *V. salmonicida*, *M. viscosa*, and IPNV (serotype sp.)	Injection	Atlantic salmon
**Coldwater Vibriosis, Furunculosis, Infectious Pancreatic Necrosis, Infectious Salmon Anemia, Vibriosis Winter Sore**	ALPHA JECT micro^®^ 7 ILA ALPHA JECT micro^®^ 7 ISA PHARMA Q	*A. salmonicida* subsp. *salmonicida*, *L. anguillarum* (serotypes O1 and O2a), *V. salmonicida*, *M. viscosa*, IPNV (serotype sp.), and ISA	Injection	Atlantic salmon
**Furunculosis, Infectious Pancreatic Necrosis, Infectious Salmon Anemia, Salmon Rickettsial Syndrome, Vibriosis**	ALPHA JECT^®^ 5-1 PHARMA Q	ISA (strain ALV301), IPNV (strain ALV103), *P. salmonis* (strain AL10005), *A. salmonicida* subsp. *salmonicida* (strain AL2017), and *V. ordalii* (strain AL510)	Injection	Atlantic Ssalmon
**Coldwater Vibriosis, Furunculosis, Infectious Pancreatic Necrosis, Vibriosis, Winter Sore**	ALPHA JECT^®^ 6-2 PHARMA Q	*A. salmonicida* subsp. *salmonicida*, *V. salmonicida*, *L. anguillarum* (serotype O1), and *M. viscosa,* and IPNV (serotype sp.)	Injection	Atlantic salmon
**Flavobacteriosis, Infectious Pancreatic Necrosis**	ALPHA JECT^®^ IPNV-Flavo 0.025 PHARMA Q	*Flavobacterium psychrophilum* (strain AL20055) and IPNV (serotype sp.)	Injection	Atlantic salmon
**Furunculosis, Infectious Pancreatic Necrosis, Salmon Pancreas Disease**	AQUAVAC^®^ PD3 MERCK-MSD	*A. salmonicida*, IPNV (serotype sp.), and SPD (strain F93-125)	Injection	Atlantic salmon
**Furunculosis, Infectious Pancreatic Necrosis, Salmon Pancreas Disease, Vibriosis, Wound Disease**	AQUAVAC^®^ PD7 MERCK-MSD	*A. salmonicida*, IPNV (serotype Sp), SPD (strain F93-125), *L. anguillarum* (serotypes O1 and O2a), *V. salmonicida*, and *M. viscosa*	Injection	Atlantic salmon
**Furunculosis, Infectious Pancreatic Necrosis, Vibriosis, Wound Disease**	AQUAVAC^®^ 6 MERCK-MSD	IPNV (serotype sp.) *A. salmonicida* subsp. *salmonicida*, *V. salmonicida*, *L. anguillarum* (serotypes O1 and O2a), and *M. viscosa*	Injection	Atlantic salmon
**Coldwater Vibriosis, Furunculosis, Infectious Pancreatic Necrosis, Winter Sore, Vibriosis**	Pentium Forte PlusTM ELANCO	IPNV (serotype sp.) *A. salmonicida* subsp. *salmonicida*, *V. salmonicida*, *L. anguillarum* (serotypes O1 and O2a), and *M. viscosa*	Injection	Atlantic salmon
**Coldwater Vibriosis, Furunculosis, Infectious Salmon Anemia, Vibriosis**	Forte^®^ VII ELANCO	ISA, *A. salmonicida*, *L. anguillarum* (serotypes 1 and 2), *V. ordalii*, and *V. salmonicida* (serotypes 1 and 2)	Injection	Salmonids
**(D) Inactivated commercially available parasitic vaccines**				
**Sea Louse disease**	Providean Aquatec Sea Lice^®^ Tecnovax https://tecnovax.com/en/product/providean-aquatec-sea-lice/	*Caligus rogercresseyi*.	Injection	Atlantic salmon

**Table 2:** List of commercially available live attenuated bacterial and viral aqua vaccines (All websites are accessed on 20 August 2024).

Disease	Vaccine	Antigen	Delivery	Species
**(A) Bacterial vaccines**				
**Salmon Rickettsial Syndrome**	ALPHA JECT LiVac^®^ SRS PHARMAQ (https://pharmaq.com/en/pharmaq/our-products)	Live culture of *Piscirickettsia salmonis* (strain AL20542)	Injection	Salmonids
**Enteric Septicemia**	AQUAVAC^®^ ESC MERCK-MSD (https://www.msd-animal-health.com/products/aquaculture/aquaculture-products/)	Live avirulent culture of *Edwardsiella ictaluri* (strain RE-33)	Immersion	Catfish
**Edwardsiellosis**	(2016) 110576037 Chinese Aquaculture Market [67]	Live bacteria culture of *Edwardsiella tarda* (strain EIBAV1)	Injection	Turbot
**Bacterial Kidney Disease**	Renogen^®^ ELANCO (https://www.elanco.com/en-us/products-and-services)	Lyophilized live culture of a microorganism that shares common antigenic determinants with *Renibacterium salmonarum*	Immersion	Salmonids
**Vibriosis**	Genetically engineered live vaccine against vibriosis (2019) Chinese Aquaculture Market [67]	Genetically attenuated live culture of *Listonella anguillarum*	Injection	Turbot
**(B) Viral vaccines**				
**Grass Carp Hemorrhagic Disease**	(2014) 190026031 Chinese Aquaculture Market [67]	Live Grass Carp Reovirus (strain GCHV-892)	Injection	Carp

**Table 3:** List of commercially available subunit bacterial and viral aqua vaccines (All websites are accessed on 20 August 2024).

Disease	Vaccine	Antigen	Delivery	Species
**Infectious Pancreatic Necrosis**	AQUAVAC^®^ IPN Oral MERCK-MSD (https://www.msd-animal-health.com/products/aquaculture/aquaculture-products/)	Recombinant Infectious Pancreatic Necrosis Virus Proteins VP2 and VP3	Oral	Atlantic Salmon
**Infectious Pancreatic Necrosis Salmon Rickettsial Syndrome**	AQUAVAC^®^ SARISTIN 2 MERCK-MSD (https://www.msd-animal-health.com/products/aquaculture/aquaculture-products/)	Recombinant Infectious Pancreatic Necrosis Virus VP2 protein and recombinant *Piscirickettsia salmonis* lipoprotein	Injection	Salmonids
**Infectious Pancreatic Necrosis Salmon Rickettsial Syndrome**	Birnagen Forte^®^ 2 ELANCO (https://www.elanco.com/en-us/products-and-services)	Inactivated culture of Infectious Pancreatic Necrosis Virus and recombinant *Piscirickettsia salmonis* HSP70, HP60, and FLG G2 proteins	Injection	Salmonids
**Edwardsiellosis Vibriosis**	(2017) 270446033 Chinese Aquaculture Market [67]	Recombinant antibodies carrying *Edwardsiella tarda* and *Vibrio* sp. idiotypes	Injection	Marine Fish

**Table 4:** Montanide ISA series used in aqua vaccine.

	Fish Species	Pathogens	References
**Montanide ISA** **-763**	Rainbow trout *Oncorhynchus mykiss*	bivalent vaccines against *Aeromonas hydrophila* and *Lactococcus garvieae*	[116]
**Montanide™ ISA 763 A VG**	Rainbow trout *Oncorhynchus mykiss*	*Yersinia ruckeri*	[117]
**Montanide ISA 760VG**	Atlantic salmon	*Flavobacterium psychrophilum*	[118]
**Montanide™ ISA 763 A VG**	Turbot (*Scophthalmus maximus* L.)	*Vibrio harveyi*	[119]
**Montanide™ ISA 763B VG and Montanide™ GEL02**	Nile tilapia (*Oreochromis niloticus*)	*Streptococcus agalactiae*	[120]
**Montanide™ ISA 763A VG and ISA 761 VG**	Rainbow trout (*Oncorhynchus mykiss*)	*Aeromonas salmonicida*	[121]
**Montanide™ ISA 763A VG and ISA 763B VG**	Nile tilapia (*Oreochromis niloticus*)	*Streptococcus agalactiae*	[122]
**Montanide™ ISA 763 AVG**	Rainbow trout (*Scophthalmus maximus* L.)	Bivalent Inactivated Vaccine against *Aeromonas salmonicida* and *Vibrio vulnificus*	[123]

**Table 5:** List of some commercially available aqua mucosal vaccines (All websites are accessed on 20 August 2024).

Disease	Vaccine Acronym	Antigen	Delivery	Species
**Vibriosis**	ALPHA DIP^®^ Vib ALPHA DIP^®^ Vibrio PHARMAQ (https://pharmaq.com/en/pharmaq/our-products)	*Listonella anguillarum* (serotype O1)	Immersion	Seabass
**Coldwater Vibriosis, Furunculosis, Vibriosis**	FORTE^®^ Micro ELANCO (https://www.elancoaquaglobal.com/us/en)	*A. salmonicida*, *L. anguillarum* (serotypes 1 and 2), *V. ordalii*, and *V. salmonicida* (serotypes 1 and 2)	Immersion	Salmonids
**Pasteurellosis**	AQUAVAC^®^ Photobac Prime MERCK-MSD (https://www.msd-animal-health.com/products/aquaculture/aquaculture-products/)	*Photobacterium damselae* subsp. *piscicida*	Immersion	Seabass, Seabream
	Icthiovac^®^ PD HIPRA	*Photobacterium damselae* subsp. *piscicida* (strains DI-21 and lt-1)	Immersion	Seabream
**Enteric Septicemia**	AQUAVAC^®^ ESC MERCK-MSD (https://www.msd-animal-health.com/products/aquaculture/aquaculture-products/)	*Live avirulent culture of Edwardsiella ictaluri (strain RE-33)*	Immersion	Catfish
**Enteric Red Mouth**	Alpha ERM Salar (Dip) PHARMAQ (https://pharmaq.com/en/pharmaq/our-products)	*Inactivated culture of Yersinia ruckeri (serotype O1b)*	Immersion	Atlantic Salmon
**Bacterial Kidney Disease**	Renogen^®^ ELANCO (https://www.elancoaquaglobal.com/us/en)	*Lyophilized live culture of a microorganism that shares common antigenic determinants with Renibacterium salmonarum*	Immersion	Salmonids
**Infectious pancreatic necrosis**	AQUAVAC^®^ IPN Oral MERCK-MSD (https://www.msd-animal-health.com/products/aquaculture/aquaculture-products/)	*Recombinant Infectious Pancreatic Necrosis Virus Proteins VP2 and VP3*	Oral	Atlantic Salmon

## Data Availability

The original contributions presented in the study are included in the article; further inquiries can be directed to the corresponding authors.
